# Environmental offsets, resilience and cost-effective conservation

**DOI:** 10.1098/rsos.140521

**Published:** 2015-07-08

**Authors:** L. R. Little, R. Q. Grafton

**Affiliations:** 1 CSIRO Oceans and Atmosphere, GPO Box 1538, Hobart, Tasmania 7001, Australia; 2 Crawford School of Public Policy, The Australian National University, Building no. 132, Lennox Crossing, Canberra, Australian Capital Territory 2601, Australia

**Keywords:** offsets, conservation budgets, resilience, ecological equivalency, substitutability

## Abstract

Conservation management agencies are faced with acute trade-offs when dealing with disturbance from human activities. We show how agencies can respond to permanent ecosystem disruption by managing for Pimm resilience within a conservation budget using a model calibrated to a metapopulation of a coral reef fish species at Ningaloo Reef, Western Australia. The application is of general interest because it provides a method to manage species susceptible to negative environmental disturbances by optimizing between the number and quality of migration connections in a spatially distributed metapopulation. Given ecological equivalency between the number and quality of migration connections in terms of time to recover from disturbance, our approach allows conservation managers to promote ecological function, under budgetary constraints, by offsetting permanent damage to one ecological function with investment in another.

## 1. Introduction

Disturbance in human-dominated environments often results in localized species extinction which can be alleviated by non-seasonal movement or migration between local populations [1]. Migration corridors that spatially connect local populations in a metapopulation vary in both quantity and quality and are known to influence recovery following a disturbance, and also resilience [2,3]. Ecological (Holling) resilience is the capacity of an ecosystem to maintain its fundamental processes and structures in the presence of shocks or change [4]. A second-order goal, typically easier to measure and implement, is the ability of a population to ‘bounce back’ as rapidly as possible following a perturbation [5]; this is commonly referred to as rapidity (Pimm) resilience.

A key challenge faced by environmental and conservation agencies is how to manage human activity and promote resilience and recovery from a negative shock in a metapopulation. Such management could involve deciding on whether to invest in either the quality or quantity of connections, and in what combination, because the benefit from each, in terms of Pimm resilience, may not be constant. A metapopulation network saturated with low-quality connections, for example, might benefit from an investment in improving the connection quality between sub-populations, while a sparsely connected metapopulation might benefit more by increasing the number of connections. At the same time, this is complicated by budget constraints, and the trade-off that any increased expenditure on the number of connections could result in fewer resources for managing the quality of connections.

Here, we examine the possible trade-off between the quality and quantity of migration connections in a metapopulation [6] in the context of Pimm resilience. We focus on the rate at which a spatially distributed metapopulation recovers from a negative shock by constructing a model, calibrated to an existing fish species in a coral reef environment. We assume that management can influence the number of migration connections between reef sub-populations, perhaps through spatial closures to fishing, particularly in the inter-reef areas, while the quality of connections is controlled, possibly through temporal protection of spawning events.

This trade-off is then cast in terms of an environmental offset. Environmental offsets are used to compensate for ecological damage at one location by protecting or repairing the expected ecological loss at another [7]. They are usually applied to biodiversity inventory and habitat, but also have been proposed for different ecological functions [8–10] by relying on the concept of ecological equivalency [11]. By introducing the concept of resilience equivalency or iso-resilience, and a budget constraint, we show that a loss or reduction in one migration factor (such as the quantity of connections) may be offset by adjusting the other factor (such as the quality of the connections). We also show how the minimum offset required to maintain Pimm resilience in the face of a permanent reduction, in either factor (quantity or quality of connections), can be calculated and measured in conservation dollars. While the approach is theoretical, and thus of general interest, it is applied to Ningaloo Reef in Western Australia to show its practical relevance.

**Figure 1.**
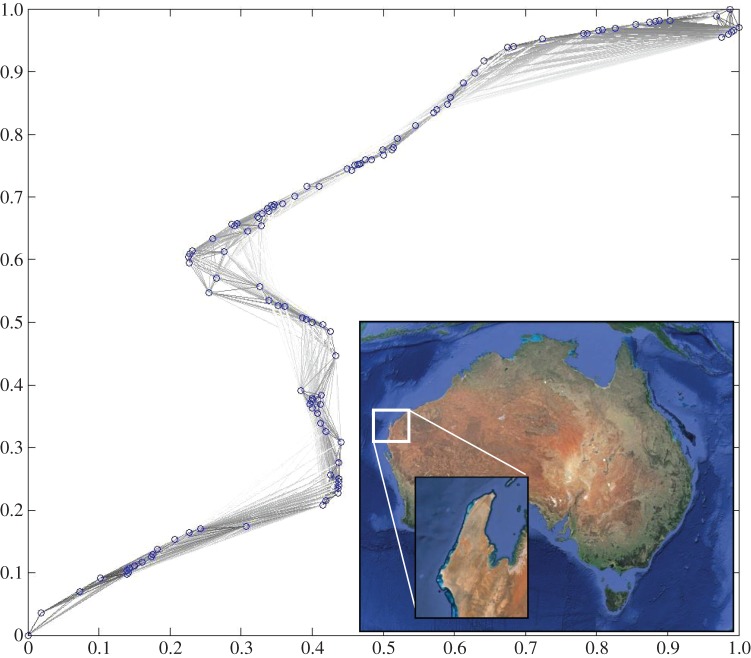
Network of 114 reef sub-populations on Ningaloo Reef, Western Australia, containing 3904 connections with link strength specified by the grey scale.

Ningaloo Reef was chosen because it is a world-heritage area approximately 300 km long in Western Australia (figure 1) and adjacent to large-scale oil and gas field developments, terrestrial mining operations and bulk shipping routes. These activities not only increase risk to the ecosystem from spills and pollution, but are also exacerbated by additional pressures from human visitation and use [12]. In response, fishery and conservation managers are required to make decisions about how to manage the reef and key species within the park boundaries.

The approach developed here provides a conceptual framework for applying offsets and managing connectivity for Pimm resilience. While applied to a specific coral reef system, the results are of general interest as they can be used in any bio-physical system where ecological equivalency can be demonstrated.

## 2. Methods and materials

A metapopulation model of a reef species, spangled emperor, *Lethrinus nebulosus*[13], was developed to explore the potential effects and responses of different network structures and migration patterns to disturbances. The model consisted of *n*=114 sub-populations linked through a connectivity matrix. The population dynamics were based on logistic growth within each sub-population, with a fraction of the sub-population production of young transferred to other sub-populations. Migration thus consisted of the productive component of the population, and not the established or settled adult component. Such migration is analogous to a settlement model for marine populations with a pelagic larval or sub-adult migration phase [14–16] with the population dynamics defined as

$$\frac{dX_{i}}{dt}=rX_{i}(1-X_{i})-srX_{i}+k_{i}\tilde{X},$$

where *X*_*i*_ is the term that defines the biomass of sub-population *i* (scaled to the carrying capacity) and *r* is the population growth rate, which represents the proportional increase in the population per unit of time (assumed to be independent of sub-population). As a result of a lack of population parameters for *L. nebulosus*, *r* was set to 0.2 based on a value found for *L. miniatus* [17], a closely related species. The other terms in this equation represent the biological (logistic) production, emigration and immigration from other sub-populations. The total amount of emigration from all sub-populations was $\tilde{X}=r\sum_{i}X_{i}$. The term *k*_*i*_ governs the distribution of emigrants among sub-populations $k_{i}=\sum_{j}L_{j,i}X_{j}/\sum_{j}L_{j,i}$, where *L*_*i*,*j*_ specifies the connectivity between sub-populations represented as the fraction of biomass that migrates from sub-population *j* to sub-population *i*. This value was calculated based on the distance between the two sub-populations *i* and *j*, which declined exponentially according to e^−3.4*D*_*i*,*j*_−3.91^, where *D*_*i*,*j*_ is the distance between two populations *i* and *j*, which was determined by fitting hydrodynamic data from a portion of the Great Barrier Reef [15] in the absence of more extensive spatially explicit connectivity data [18,19]. Only values of the expression e^−3.4*D*_*i*,*j*_−3.91^ that were greater than 0.01 were used so as to constrain the size of the network, which resulted in a network of 3904 links. For the purpose of this paper, we modified the expression by scaling it to the connection quality *s*, so that *L*_*i*,*j*_=*s*×e^−3.4*D*_*i*,*j*_−3.91^. The coefficient *s* (where 0≤*s*≤1) represented the quality of connection between sub-populations as the survivorship of individuals undertaking migration.

### 2.1 Simulations

The metapopulation was distributed in the Cartesian plane (figure 1), with sub-populations assigned according to the distribution of reefs used by Thébaud *et al.*[13]. Disturbances to the metapopulation were imposed in the model by removing 99% of the biomass from each sub-population within 0.4 units from the centre of a point selected randomly in the spatial domain based on uniform random numbers on the *x*- and *y*-axes. Different numbers of connections between sub-populations, *α*, were determined from random selection (without replacement) of the 3904 links calculated above.

We investigated the effect of having a different number of connections, defined by the *α* parameter, combined with different quality of connections, defined by the *s* parameter, which specifies the amount of productive capacity that is transferred in the migration network. For each of 11 values of *s* between 0 and 1, and 10 values of *α* between 200 and 3904 we replicated 10 000 Monte Carlo simulations (a minimum of 200 links gave each sub-population roughly one link each), so that each had a different spatial location of disturbance and a different configuration of *α* connections.

The modelled population trajectories were solved using a Runge–Kutta numerical procedure. The biomass *X*_*i*_ (relative to carrying capacity) through time was summed across sub-populations, and the recovery time, or Pimm resilience, measured as the number of time steps taken for the entire metapopulation to recover 95% of its initial state.

Mean recovery times across the Monte Carlo simulations were shown graphically as a contour plot with the value of connection quality, *s*, and quantity, *α*, on the *x*- and *y*-axes, respectively. The contours showed the trade-off between *s* and *α*, while maintaining a constant recovery time (resilience). By introducing on the same plot the cost trade-off between spending on connection quality and quantity under a given budget, we showed how investment in connection quality *s*, through an increased budget, can compensate or offset the loss in the number of connections *α*.

We conducted a sensitivity analysis to show which parameter choices and aspects of the model influenced model responses. We increased and decreased the population growth rate *r* (0.25 and 0.15) as well as added a reef-specific population growth rate *r*_*i*_, such that it increased (or decreased) according to reef latitude, e.g. *r*_*i*_=0.2+lat_*i*_−mean(lat_*i*_), for reef *i*, latitude lat_*i*_, while maintaining the average across *r*_*i*_ as 0.2. Results were also shown for model scenarios where disturbance only reduced the affected populations by 50%, where there are no localized effects of migration (*L*_*i*,*j*_=1,∀*i*,*j*), and where there is increased localization of sub-populations (i.e. *L*_*i*,*j*_=*s*×e^−6.8*D*_*i*,*j*_−3.91^).

## 3. Results

**Figure 2.**
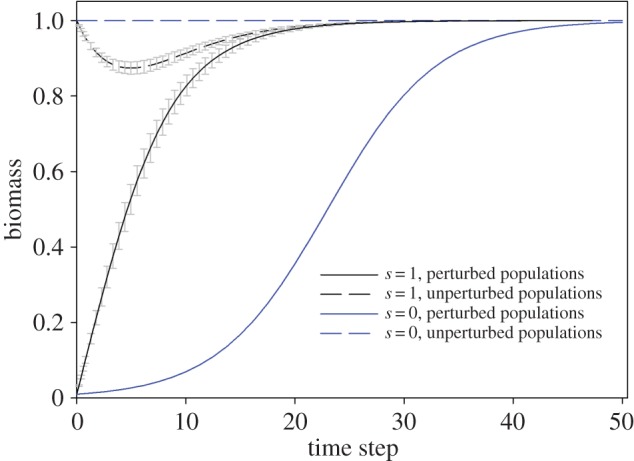
Average time trajectory (±s.d.) of 10 000 replicate Monte Carlo model recovery paths of perturbed and unperturbed sub-populations connected in the full migration network (*s*=1) of 3904 connections, or independent of each other (*s*=0).

Sub-populations in the disturbance area had a faster rate of recovery if they were connected to sub-populations outside of the disturbance area (figure 2). This result is analogous to an earlier finding that connectivity may be important in species persistence within reserve networks [20]. This had an effect on the undisturbed sub-populations (black dotted line, figure 2) as they supported the recovery of the disturbed sub-populations.

The effect of different model parametrization scenarios indicated that recovery time decreased with a larger population growth rate, *r* (table 1). The spatial structure of the metapopulation had an observable effect on the ability to recover from disturbance. Recovery times were longer when the population growth rate was highest in the south, owing perhaps to greater isolation of the southern reefs. A smaller disturbance of 50% removal of sub-populations rather than 99% led to faster recovery times. Changing the spatial effect of migration affected recovery times when the quality of connection was high, *s*=1.0, but not at all when the quality of connections was poor or absent, i.e. *s*=0.0. Specifically, under *s*=1.0 and when all links *L*_*i*,*j*_ had the same value (1.0) irrespective of distance, the recovery time declined to 17.1 from 25.8 time steps, as there was no penalty for migration between reefs at the northern and southern extremes. When sub-populations were more localized (twice spatial effect on migration) recovery times increased to 29.3 time steps, but were still less than if there was no connections (*s*=0.0) which recovered in 33.9 time steps (table 1).

**Table 1.** Average (±s.d.) recovery time (resilience) under a range of model scenarios each with 3904 connections, and two levels of *s* (0.0 and 1.0).

scenario	*s*: 1.0	*s*: 0.0
base case	25.8 ± 3.3	33.9 ± 2.4
*r* = 0.25	20.6 ± 2.7	27.2 ± 1.8
*r* = 0.15	34.4 ± 4.5	45.2 ± 3.0
disturbance = 50%	10.6 ± 2.1	11.1 ± 2.4
no spatial effect on migration (*L*_*i*,*j*_=1,∀*i*,*j*)	17.1 ± 3.3	33.9 ± 2.3
twice spatial effect on migration (*L*_*i*,*j*_=exp(−6.8*D*_*i*,*j*_−3.91))	29.3 ± 2.8	33.9 ± 2.3
*r* northerly increase	26.1 ± 3.5	35.2 ± 4.6
*r* southerly increase	28.5 ± 4.7	38.0 ± 4.7

**Figure 3.**
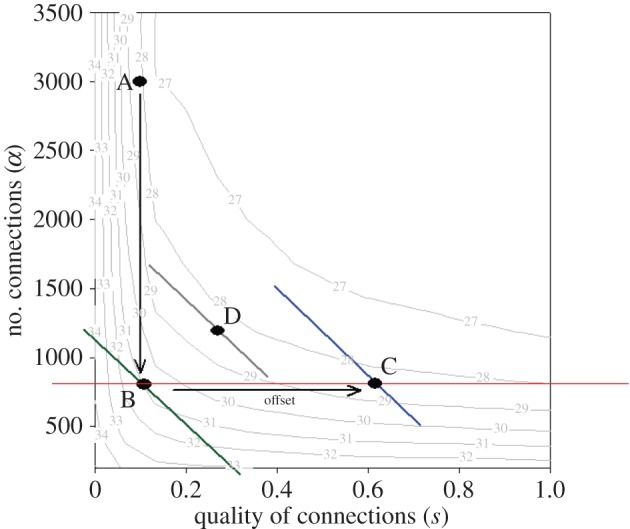
Iso-recovery/resilience curves of average recovery time for different combinations of connection quantity and quality (*α* and *s*). The green, grey and blue lines represent different budget constraints. The blue line (at point C) represents a higher budget than all others while the green budget line (at point B) has the lowest conservation budget. In the absence of any limits on either the number or quality of connections, the fastest possible recovery time for a given budget is where the budget line is tangent to the iso-resilience curve furthest from the origin (e.g. point D on the grey budget line).

Pimm resilience is shown for the full combination of *α* and *s* scenarios in terms of an indifference map of iso-recovery or iso-resilience lines (figure 3) at specific intervals, which represent identical recovery times for different combinations of *α* (number of connections) and *s* (quality of connections). The greater the number of connections, for a given connection quality, the faster the population recovered following a disturbance. This occurred at a decreasing rate. Increasing the quality of the connections (*s*), i.e. the amount of migration between sub-populations, for a fixed number of connections reduced the recovery time, but again at a decreasing rate.

A hypothetical fiscal budget available to be invested in reserve networks can be introduced as a budget line (green line, figure 3). The total budget is represented by the intercepts of the line with the axes, and the budget constraint for management is the choice of *α* and *s* along a line that can be afforded under a specific budget. In the marine context, connectivity, *s*, might be controlled by expenditure on temporal spawning closures that protect spawning aggregations from fishing disruption [21] while the number of connections between sub-populations, *α*, could be managed through permanent spatial closures to fishing, particularly activities in the inter-reef areas [22].

The linear budget line is a result of assuming that there is a fixed marginal cost in the number of connections (*α*) in terms of the cost of connectivity (*s*). A change in cost that varied with either the quality or the number of connections, or both, would imply a nonlinear budget constraint, which can be solved to determine the highest iso-resilience curve for a given conservation budget. The minimal recovery time for a given budget, in the absence of constraints on the number and quality of connections in the reserve network, occurs when the slope of the budget line equals, i.e. tangent to, the slope of the iso-resilience curve (point D for the grey budget constraint, figure 3 with a numerical value of 28.5).

Figure 3 shows a hypothetical shift from an original state of nature at point A to B as the result of a permanent change in human activity, such as trawling the inter-reef [22] or nursery habitat, or on-going human activity, such as disturbances from oil and gas developments or bulk shipping, that reduce the number of connections, but not the quality of those connections remaining. Thus, an important condition of this illustration is that the disturbance is highly localized. The least cost offset from point B to maintain the previous level of Pimm resilience or recovery time (28.5) would be at point D on the grey budget line, but this would require more metapopulation connections which are not possible given the human activity.

If, in figure 3, the maximum number of connections is limited to 800 at point B, then the minimum conservation budget in dollar terms needed to return to the level of resilience at A (28.5) is represented on the blue budget line at point C. The conservation offset required to return the system to its previous level of Pimm resilience (value =28.5) is achieved by improving the quality of the remaining connections from *s*=0.12 to 0.62 and is represented by the difference between the blue budget line (at point C) and the green budget line (at point B). In other words, the *minimum* offset, in dollar terms, which is needed to ensure the same speed of recovery while restricted to a maximum number of connections at 800 (red line), is the change in the quality of connections from *s*=0.12 to 0.62.

Improvements in the quality of connections to offset the reduction in the quantity of connections could arise from various conservation actions such as temporal closures to fishing that prevent disruption of spawning events or possibly changes in where oil and gas exploration takes place. A less costly budget could attain the level of resilience at point D, but would require more connections (greater than 800) which, in this illustration, is not feasible given the human activity. Thus, a second important condition or assumption in this example is that the management response affects only the connection quality. Any interaction that affected the number of connections would correspondingly result in either more or less resilience.

## 4. Conclusion

Our model showed how conservation networks of sub-populations can respond to disturbance and how the network characterization of the quantity and quality of connections between sub-populations affects the response to disturbance, in terms of recovery time. The results showed: (i) that an increase in both the number of connections and their quality reduced recovery time (increased Pimm resilience) and (ii) that the number and quality of connections may, within some range, be substituted to achieve a given speed of recovery. This substitution we believe forms a conceptual basis for environmental offsets.

Environmental offsets attempt to prevent or to reduce a net environmental loss resulting from human activities by compensating for the damage made to one ecological asset by investing in or protecting another [7]. The effectiveness of offsets relies on the assumption of substitutability [23], and they have been typically examined in terms of habitat. We showed the substitutability of two metapopulation characteristics, migration connectivity (quantity) and migration capacity (quality), and the financial or budgetary offset needed to restore or compensate the ecological function of recovery from a stochastic disturbance (Pimm resilience).

Framing the trade-off between connection quantity and quality on an iso-resilience curve (or an indifference map) provides a baseline of comparison for encountering a permanent disturbance [24] and shows how conservation managers can calculate an offset, measured in conservation dollars, to compensate for the loss in a key ecosystem function, Pimm resilience. While ecosystem function encompasses much more than Pimm resilience, iso-resilience curves could offer a cost-effective way to promote long-term protection of biodiversity.

Our method allows conservation managers to calculate the *minimum*compensation needed to achieve a similar ecosystem function given a permanent disruption. While we showed this for a single species metapopulation, there are potentially many species affected, and compensation to restore Pimm resilience to other parts of the ecosystem may not respond in the same manner. This is often the way environmental offsets are implemented in practice. Nevertheless, the focus on a single species may be warranted if it is threatened, endangered or specifically protected.

The application of iso-resilience curves to decisions about offsets assumed that both the number and quality of connections were subject to effective management control [25,26]. The degree to which the quality of connections between sub-populations, represented as *s*, is under management control is likely to depend on the life history of the species concerned, and also on the matrix in which the metapopulation is embedded. Arguably, the hydrodynamic complexity may compound the difficulty of applying our methods to a reef system.

A simple model was developed for a metapopulation subject to spatially random disturbances which, as far as possible, included the dynamics of an actual fish population. Nevertheless, fully incorporating all the stochastic elements of the population and environmental disturbances remains a challenge. Our method is valuable because although conservation planning tools intended to design reserve networks are well developed [27,28], with a large degree of realism, the concepts, methods and tools used to support environmental offsets are much less effectively represented.

One of the key goals in environmental offsetting is conserving species and habitats. An offset is usually viewed as a separate and exogenous, but an equivalent area to one that is subject to human-mediated disturbance. We adopted a somewhat different perspective and calculated how an ecological function can be endogenously maintained, or offset. This was done by representing an ecological function, namely Pimm resilience of a population, on an indifference map with a choice between two ecological factors, the quantity and quality of migration connections, which not only affect the ecological function, but also can be affected by management intervention. Using this approach, ‘cost’ can be valued either monetarily, as we have done, or in terms of another currency either as a ‘debit’ or ‘credit’ for conservation banking purposes [29,30].

Our approach offers a potential method for cost-effective conservation decision-making, where disruption from human activities is unavoidable. By maximizing resilience in response to anthropogenic disturbance, while also accounting for a limited conservation budget, management agencies should be better able to opportunistically to respond to natural disturbances and generate greater ecosystem services [31].

## Supplementary Material

Supplementary Material to Environmental offsets.docx

## Supplementary Material

Supplementary Material_ReefLinkData_Ningaloo.txt

## Supplementary Material

Supplementary Material_ReefNodeLocation_Ningaloo.txt

## Data accessibility

Data available at http://catalogue.aodn.org.au/geonetwork/srv/eng/metadata.show?uuid=bc1b3741-e7e6-5039-e044-00144f7bc0f4 and in the electronic supplementary material.
