# Supplementary material for: Environmental offsets, resilience and cost-effective conservation
Source: R Soc Open Sci. 2015 Jul 8;2(7):140521. doi: 10.1098/rsos.140521 (PMC4632573; doi:10.1098/rsos.140521)
Supplement: Supplementary Material to Environmental offsets.docx [file rsos140521supp1.docx]

Supplementary Material to Environmental offsets, resilience and the cost-effectiveness of conservation

Matlab model code:

function dy = metanetwork4(t,y,n,s,r,L)

dy = zeros(n,1); % a column vector

yprod = sum(r .* y);

I = L * y;

% to avoid NaNs when there is a 0 just set it to 1 for the denom

denom = sum(L,2);

denom(denom == 0) = 1;

I = I ./ denom; % this is the total arriving at the each location i

I = I / sum(I);

for k=1:n

dy(k) = r(k) * y(k) * (1 - y(k)) - (1 - s) * y(k) * r(k) + (1 - s) * yprod * I(k);

end;

close all

clear all

r = 0.2;

perturbAmount = 0.01;

disturbanceRadius = 0.4;

s = 1.0;

%s = 0.0;

absoltol = 1e-4 *ones(1,n);

options = odeset('RelTol',1e-4,'AbsTol',absoltol);

% read reef location data

fid = fopen('./ReefNodeLocation_Ningaloo.txt', 'r');

mydat = textscan(fid, '%d %f %f');

fclose(fid);

nindex = mydat{3}(:);

x = mydat{2}(:);

z = mydat{3}(:);

n = size(nindex, 1);

nodes = [nindex x z];

% read reef link data

fid = fopen('./ReefLinkData_Ningaloo.txt', 'r');

linkdat = textscan(fid, '%d, %f, %f');

fclose(fid);

src=linkdat{1}(:);

snk=linkdat{2}(:);

lnk=linkdat{3}(:);

nLinks = size(src,1);

LplotX = zeros(2,nLinks);

LplotZ = zeros(2,nLinks);

LLz = zeros(nLinks,1);

% initialise Links between sub-populations

Lbase = zeros(n,n);

for i=1:nLinks

Lbase(src(i)+1,snk(i)+1)=lnk(i);

LplotX(1,i) = x(src(i)+1);

LplotX(2,i) = x(snk(i)+1);

LplotZ(1,i) = z(src(i)+1);

LplotZ(2,i) = z(snk(i)+1);

LLz(i) = lnk(i);

end

fprintf( ' %d nodes, with %d links \n', n,nLinks);

L = zeros(n,n);

L = Lbase;

% initialise sub-populations

y0 = ones(1,(n));

% random disturbance affects only some of the sub-populations

k = round(rand(1)*(n-1))+1;

perturb_x = x(k);

perturb_z = z(k);

for i=1:n

dist2centre = sqrt((x(i)-perturb_x)^2 + (z(i)-perturb_z)^2);

if dist2centre <= disturbanceRadius

y0(i) = perturbAmount;

end;

end;

% solve model

[T,Y] = ode45(@(t,y)metanetwork4(t,y,n,s,r,L),[0 Tmax],y0,options);

Data:
